# Investigation of a Novel Ultra-Low-Frequency Rotational Energy Harvester Based on a Double-Frequency Up-Conversion Mechanism

**DOI:** 10.3390/mi14081645

**Published:** 2023-08-20

**Authors:** Ning Li, Hu Xia, Chun Yang, Tao Luo, Lifeng Qin

**Affiliations:** 1 Shenzhen Research Institute of Xiamen University, Shenzhen 518000, China; 19920221151543@stu.xmu.edu.cn (N.L.); frighten@stu.xmu.edu.cn (H.X.); 19920211151546@stu.xmu.edu.cn (C.Y.); luotao@xmu.edu.cn (T.L.); 2 Department of Mechanical and Electrical Engineering, Xiamen University, Xiamen 361005, China

**Keywords:** piezoelectric, energy harvesting, rotational, ultra-low frequency

## Abstract

Due to their lack of pollution and long replacement cycles, piezoelectric energy harvesters have gained increasing attention as emerging power generation devices. However, achieving effective energy harvesting in ultra-low-frequency (<1 Hz) rotational environments remains a challenge. Therefore, a novel rotational energy harvester (REH) with a double-frequency up-conversion mechanism was proposed in this study. It consisted of a hollow cylindrical shell with multiple piezoelectric beams and a ring-shaped slider with multiple paddles. During operation, the relative rotation between the slider and the shell induced the paddles on the slider to strike the piezoelectric beams inside the shell, thereby causing the piezoelectric beams to undergo self-excited oscillation and converting mechanical energy into electrical energy through the piezoelectric effect. Additionally, by adjusting the number of paddles and piezoelectric beams, the frequency of the piezoelectric beam struck by the paddles within one rotation cycle could be increased, further enhancing the output performance of the REH. To validate the output performance of the proposed REH, a prototype was fabricated, and the relationship between the device’s output performance and parameters such as the number of paddles, system rotation speed, and device installation eccentricity was studied. The results showed that the designed REH achieved a single piezoelectric beam output power of up to 2.268 mW, while the REH with three piezoelectric beams reached an output power of 5.392 mW, with a high power density of 4.02 μW/(cm^3^ Hz) under a rotational excitation of 0.42 Hz, demonstrating excellent energy-harvesting characteristics.

## 1. Introduction

With the widespread adoption of 5G networks and the rapid development of Internet of Things (IoT) technologies, numerous electronic devices have been applied in various corners of the world. However, the increasing demand for power supply among these electronic devices poses a challenging problem. Traditional battery-powered solutions for electronic devices working in complex and harsh environments, such as various sensors, suffer from issues like short lifespans, high replacement costs, and environmental pollution after disposal. Therefore, there is an urgent need for a new type of power device that can provide a long-term energy supply. Energy harvesters, as emerging energy supply devices, leverage various mechanisms, such as piezoelectric [1,2,3], electromagnetic [4,5], triboelectric [6,7], and photovoltaic [8,9] effects, to convert mechanical energy, wind energy, solar energy, and other forms of ambient energy into electrical energy for external output. Among them, piezoelectric energy harvesters [10,11,12] have been employed in widespread applications due to their simple structure [13,14], long lifespan [15,16], and high adaptability [17,18,19] to environmental conditions. These devices operate by utilizing the mechanical energy obtained from the external environment to induce deformation in the internal piezoelectric material. Subsequently, the piezoelectric effect of the material is harnessed to convert the collected mechanical energy into electrical energy and output it externally. Depending on the type of motion in the application environment, such as vibration [20,21], oscillation [22,23], and rotation [24,25], piezoelectric energy harvesters can be classified as vibration energy harvesters [26,27] and rotational energy harvesters [28,29,30]. Extensive progress has been made in the research on vibration-based energy harvesters. Meanwhile, although it lags behind research into vibration-based energy harvesters, in order to meet the growing demand for energy conversion and utilization, research in the field of rotation-based energy harvesters is also rapidly advancing.

In the field of piezoelectric rotational energy harvesters (REHs), three types can be identified based on the mode of forced deformation of the piezoelectric material: magnetic-driven [31,32], gravity-driven [33,34], and mechanical-plucking-driven [35,36,37] REHs. For instance, in the work by Zou et al. [38], researchers employed a disc rotating around the center with magnets placed on its circumference as the rotor, while a bridge-like structure containing piezoelectric material and magnets was fixed around the disc as the stator. During the operation of the REH, as the magnets on the rotor approached the magnets on the stator, the bridge-like structure deformed under magnetic forces, thereby inducing compression and tension on the internal piezoelectric material for energy conversion. However, the use of magnetic-driven REHs is limited when the application environment is sensitive to magnetic fields. Conversely, REHs that utilize gravity for external excitation to generate the output can effectively avoid this issue. In the work by Guan et al. [39], researchers fixed one end of the piezoelectric beam on the rotor, while another end had a mass block attached, with the center of gravity aligned with the rotation center. During the rotation of the REH, the periodic deformation of the piezoelectric beam was driven by the gravitational force, converting mechanical energy into electrical energy. The results demonstrated that the REH achieved a power density of 0.81 μW/(cm^3^ Hz) at an excitation frequency of 0.79 Hz. However, due to the gravitational excitation period matching the system’s rotational period, the output performance of the REH was limited in ultra-low-frequency rotational environments. In contrast, the mechanical-plucking approach often allows the piezoelectric beam to undergo self-excited oscillation at its inherent frequency after being mechanically plucked, thereby enhancing the output capability of the REH in low-frequency rotational environments. In the work by Fang et al. [40], researchers designed a rotating energy harvester composed of a cylindrical rotor with multiple plucking elements and a stator with multiple piezoelectric beams. Similar to the mechanism of a music box, when the REH was in operation, the motion of the rotor at the rotation center triggered the plucking elements to strike the piezoelectric beams, inducing self-excited oscillation for energy conversion. However, this fixed-rotor or stator arrangement necessitated mounting one of them at the rotation center. In certain rotational environments where the installation of external mechanisms is impractical due to structural constraints (e.g., car wheels) or where the devices requiring powering are located far from the rotation center, the application of such REHs is limited. To address such issues, in our previous work [41], we designed and fabricated an REH that utilized liquid as the energy-capturing medium. The proposed REH included a piezoelectric beam with a flow resistance plate, a cylindrical shell, and a liquid. The fluid’s flow characteristics enabled it to impact the piezoelectric beam at low rotational speeds and act as a mass block at high rotational speeds, inducing the periodic deformation of the internal piezoelectric material and generating an electric output based on the piezoelectric effect. The results demonstrated that the REH achieved a power density of 0.23 μW/(cm^3^ Hz) at an excitation frequency of 1.25 Hz. However, this was still insufficient for effectively powering devices with higher energy demands. In conclusion, although significant progress has been made in the research on rotational energy harvesters, achieving higher power outputs in low-frequency rotational environments (<1 Hz), such as wind turbines and hydro turbines [13,41], remains a challenging issue to be addressed.

In this study, we proposed a novel ultra-low-frequency rotational energy harvester based on a double-frequency up-conversion mechanism. The harvester consisted of a cylindrical shell internally equipped with multiple piezoelectric beams and a slider with multiple paddles attached. During the movement of the rotational energy harvester (REH) along with the rotating system, a relative rotation occurred between the slider and the shell due to the influence of gravity, resulting in the activation of the paddles to strike the piezoelectric beams, inducing the self-excited oscillation of the beams (the first frequency up-conversion) and enabling the conversion of mechanical energy into electrical energy through the utilization of the piezoelectric effect. Simultaneously, by appropriately increasing the number of paddles on the slider and the number of internal piezoelectric beams in the REH, a significant enhancement in the collision frequency between the paddles and piezoelectric beams within one rotation cycle could be achieved (the second frequency up-conversion), thereby further improving the output performance of the REH. During the experiment, the proposed REH achieved an external output power of 5.392 mW and a power density of 4.02 μW/(cm^3^ Hz) under a rotational excitation of 0.42 Hz. Additionally, the proposed design of the REH allowed for the retention of the relative motion between the internal slider and shell even when the REH was eccentrically installed away from the rotational center of the system. Therefore, under the conditions of eccentric installation, the REH remained capable of harvesting rotational energy. The paper is organized as follows: Section 2 discusses the working principle of the proposed energy harvester. Section 3 presents the fabrication process and experimental setup. Section 4 provides a detailed analysis of the results and includes a comprehensive discussion. Finally, Section 5 presents the meaningful conclusions drawn from the findings.

## 2. Working Principle of Proposed REH

**Figure 1:**
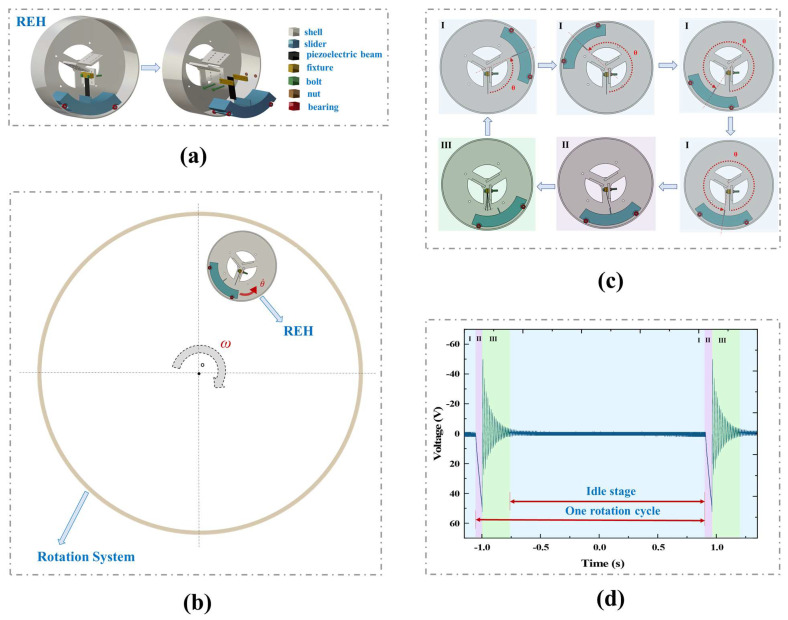
Operating principle of the REH: (**a**) composition of the REH; (**b**) REH in the rotating system; (**c**) operating principle of the REH; (**d**) open−circuit voltage waveform of the REH (system rotation speed *ω* = 30 rpm, number of paddles *n* = 1, number of piezoelectric beams *k* = 1).

As shown in Figure 1a, the proposed REH consisted of a cylindrical shell and a slider with paddles. Inside the cylindrical shell, there was a clamping platform designed to hold the piezoelectric beams. As shown in Figure 1b, the proposed REH was installed in the rotational system and rotated together with the system. Figure 1c illustrates the relative motion between the internal slider and the shell of the REH during this process. Simultaneously, we captured the open-circuit voltage waveform output by the REH within one rotation cycle using an oscilloscope, as shown in Figure 1d. Combining Figure 1c,d, it can be observed that the complete output process of the device in each rotation cycle consisted of three stages. The first stage occurred when the slider approached the piezoelectric beams, at which point the piezoelectric beams had no external output. The second stage was the contact between the paddles and the piezoelectric beams, where the piezoelectric beams rapidly reached their maximum deformation under the pushing force exerted by the paddles on the slider. Subsequently, the process entered the third stage, where the paddles detached from the piezoelectric beams, and the piezoelectric beams underwent self-excited oscillation with their inherent frequency (the first frequency up-conversion). It was in this stage that the mechanical energy was converted into electrical energy by the piezoelectric effect [42]. However, in an ultra-low-frequency rotational environment (<1 Hz), there is a long idle period between collisions due to the longer rotation period, during which the piezoelectric beam remains idle and does not generate an output. This phenomenon was also confirmed by the waveform of the output voltage obtained from the experiments, as shown in Figure 1d, which demonstrated a long interval between adjacent waveforms, indicating the significant proportion of the collision process taken up by the idle stage. This was caused by the longer rotation period at low frequencies and had a negative impact on the output performance of the REH.

**Figure 2:**
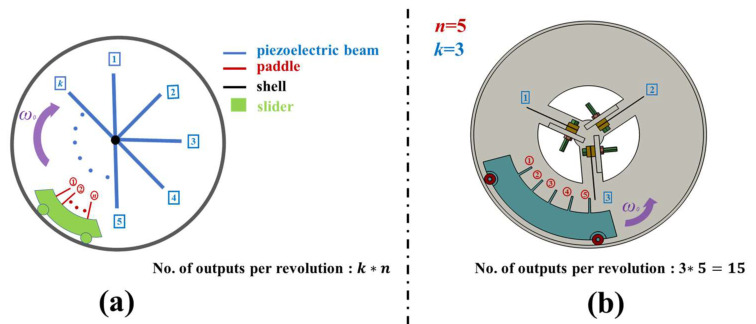
Prototype of the proposed REH (the numbers in the red labels correspond to the paddle indices, while the numbers in the blue labels correspond to the piezoelectric beam indices): (**a**) proposed energy harvester concept; (**b**) schematic drawing of the proposed REH (number of piezoelectric beams *k* = 3, number of paddles *n* = 5).

Therefore, in order to enhance the output performance of the proposed REH, our goal was to minimize the proportion of the “idle stage” during the operation of the REH. We proposed a feasible solution to achieve a reduction in the “idle stage” by increasing the number of paddles attached to the slider, thereby increasing the frequency of the piezoelectric beams being actuated within one rotation cycle. Additionally, by increasing the number of piezoelectric beams inside the REH, the number of times the beams were actuated within one rotation cycle could also be increased. Therefore, when the REH contained *k* piezo beams and *n* paddles, the number of external outputs per rotation cycle reached k×n (the second frequency up-conversion), as shown in Figure 2a (for example, when *n* = 5 and *k* = 3, the REH obtained is shown in Figure 2b).

**Figure 3:**
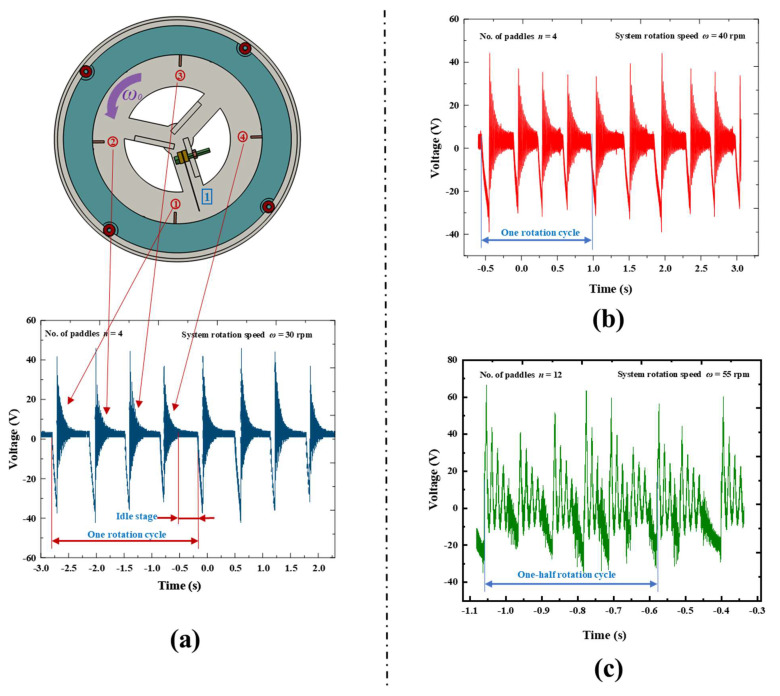
Open−circuit voltage waveforms of the REH at various rotational speeds and numbers of paddles (number of piezoelectric beams *k* = 1, the numbers in the red labels correspond to the paddle indices, while the numbers in the blue labels correspond to the piezoelectric beam indices.): (**a**) system rotation speed *ω* = 30 rpm, number of paddles *n* = 4; (**b**) system rotation speed *ω* = 40 rpm, number of paddles *n* = 4; (**c**) system rotation speed *ω* = 55 rpm, number of paddles *n* = 12.

In order to validate the feasibility of the design concept, we conducted preliminary experiments using an REH equipped with a single piezoelectric beam. The REH-4 (an REH with four attached paddles, *n* = 4) was employed for testing, and the obtained waveforms are depicted in Figure 3a. Under the same rotational speed (30 rpm), as the number of paddles increased, the time interval between adjacent waveforms decreased. This indicated a higher proportion of effective working time for the REH within an ultra-low-frequency rotational environment. Therefore, increasing the number of paddles was a feasible approach to enhance the output performance of the proposed REH. Additionally, we tested the output waveforms of the device with different numbers of paddles and higher system rotational speeds, as shown in Figure 3b,c. From the figures, it can be observed that interference occurred between adjacent waveforms when the number of paddles or rotational speed exceeded a certain threshold. The impact of this interference on the REH’s performance was further investigated in subsequent experiments.

To further understand the working mechanism of the proposed REH, we needed to perform a dynamic analysis of its motion process. However, a similar dynamic model was established in our previous work [41], so we only provide a brief introduction to the establishment of the dynamic model in this paper.

**Figure 4:**
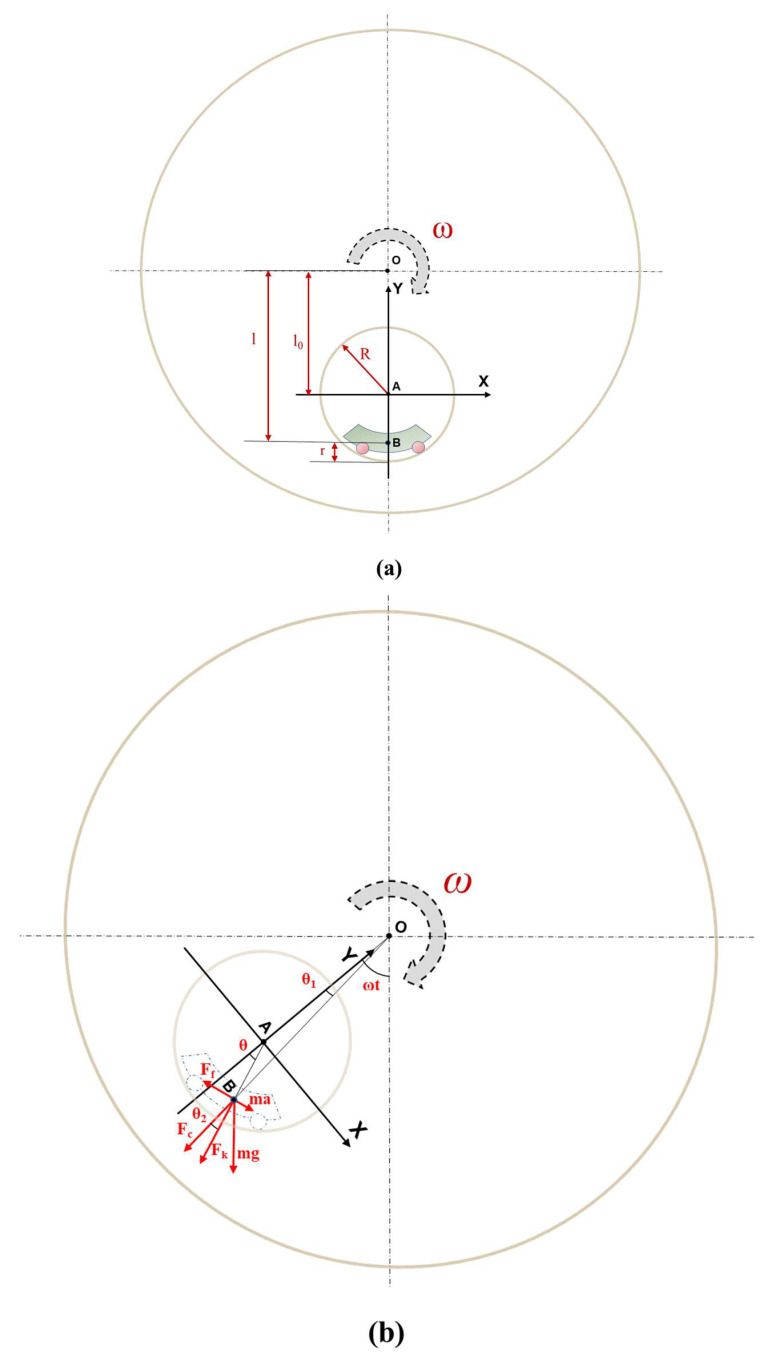
Dynamic model illustration of the REH operation process: (**a**) dimensional parameters of the system; (**b**) dynamic parameters of the system.

Prior to the dynamic analysis, we simplified the model. Firstly, since the collision between the paddles and the piezoelectric beams was an instantaneous loading and unloading process, which depended on factors such as the mass of the slider and the motion state of the rotational system, it was a complex action process. Therefore, the influence of this process on the theoretical modeling was not considered temporarily in the dynamic analysis. Secondly, because the motion states of different parts of the slider were relatively consistent during its movement, and the shape of the slider had a minor impact on the motion process, the dynamic analysis could adopt a point mass to represent the slider, with the position of the point mass coinciding with the center of mass of the slider. On this basis, kinetic parameters were introduced into the system. As shown in Figure 4, the proposed REH was installed on a rotational system with angular velocity *ω* and an eccentricity distance of *l*_0_ (the distance from the center of the rotation system (point O) to the center of the cylindrical shell (point A)). A relative coordinate system XAY was established inside the REH, and the relevant parameters are labeled accordingly. Meanwhile, specific definitions of the parameters are provided in Table 1.

Building upon this foundation, we employed the same modeling approach as in our previous work [41] to obtain the final results from the dynamic analysis.

In the relative coordinate system (XY) with the origin located at the center (point A) of the cylindrical shell and synchronized with the rotational system, the dynamic equation for the slider could be obtained through the analysis of the motion process as follows:

(1) $$m\ddot{\theta }(R-r)=mg\sin\left(\omega t-\theta \right)-F_{c}\sin\theta_{2}-\mu (m\omega^{2}l\cos\theta_{2}+mg\cos\left(\omega t-\theta \right)+2m\omega v)$$

where *μ* is the friction coefficient. Meanwhile, the relationship between *θ* and *θ*_2_ could be expressed as

(2) $$\theta =\sin^{-1}\left(\frac{OA}{AB}\sin\theta_{2}\right)+\theta_{2}$$

**Figure 5:**
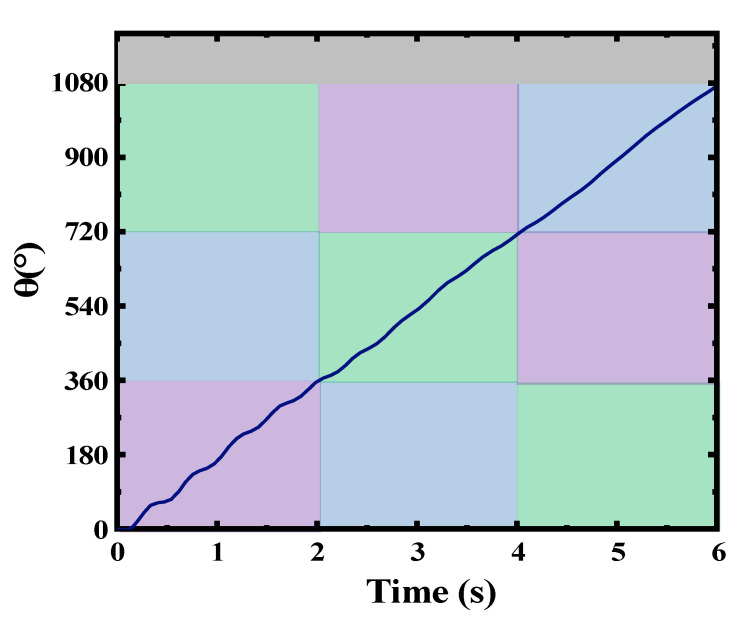
Displacement of the slider with time in angular motion.

Based on Equations (1) and (2), the variation in the relative angular displacement between the center of mass of the slider and the shell with time could be obtained. Subsequently, the MATLAB Simulink module could be used to simulate the variation in the relative angular displacement with time. The simulation results are shown in Figure 5. Under low-speed conditions (taking the system speed as 30 rpm, *l*_0_ = 120 mm, *R* = 53 mm, and *r* = 20 mm), the angular displacement *θ* of the slider increased with time, while the angular velocity $\dot{\theta }$ of the slider remained nearly constant during the rotation of the system. This phenomenon was also verified in practical experiments. Based on the analysis above, we could draw the conclusion that at lower system rotation speeds (<1 Hz), the relative rotational speed between the slider and the shell remained stable and numerically equal to the system’s rotation speed. This result was also verified in subsequent experiments, as depicted by the waveforms in Figure 3.

## 3. Fabrication and Experimental Setup

**Figure 6:**
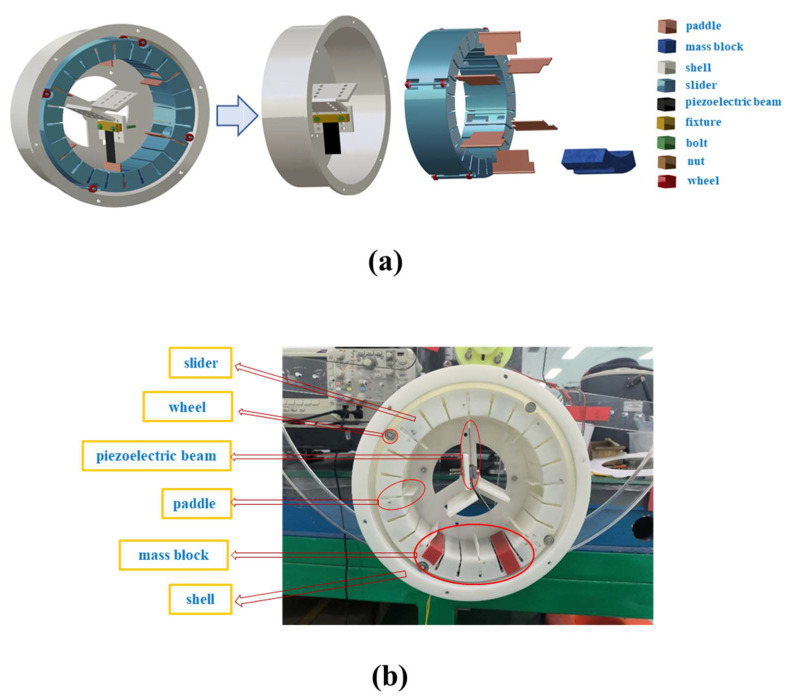
Prototype of the REH used in the experiments: (**a**) internal structure of the REH; (**b**) physical prototype of the proposed REH.

In order to verify the feasibility of the proposed REH for effective energy harvesting in low-speed environments, a prototype was fabricated as shown in Figure 6b. It consisted of a hollow cylindrical shell and an internally hollow cylindrical slider, with the internal structure depicted in Figure 6a. Within the cylindrical shell, three holding platforms with an angular spacing of 120° were designed. Piezoelectric beams were installed on these platforms using fixtures and fasteners, allowing for adjustable displacement along the arrangement direction of the through holes on the platforms. As for the cylindrical slider, it featured four wheel shafts with an angular spacing of 90° on the outer side, along with four sets of bearings to reduce the frictional force between the slider and the shell. Simultaneously, the inner side of the slider contained 24 slots with an angular spacing of 15°, and six cavities with an angular spacing of 60° were designed within the slider using partition plates. The slots were utilized for mounting paddles. At the same time, the bottom of each paddle was designed in a cylindrical shape to match the array of through holes at the bottom of the slots, thereby adjusting the distance between the top of the paddle and the center of the shell. The number of paddles and the angular spacing between them could be adjusted by matching the slots with the paddles. Furthermore, the cavities were used to accommodate weights, ensuring that the mass center of the slider was positioned at the lower end, thus guaranteeing the stability of relative motion between the slider and the shell. The mass of the weights is represented as *G*.

The shell, slider, and paddles were all produced via 3D-printing technology, utilizing UV-curable resin material (SOMOS Imagine 8000, Royal DSM Group, Heerlen, the Netherlands). The cylindrical shell had a diameter of 231 mm, a height of 76 mm, and an inner-wall thickness of 2 mm. The piezoelectric cantilever beam (PZT 5J S118-J1SS-1808YB) in the device was produced by Mide Technology Company in the United States,, with detailed parameters provided in Table 2. The outer dimensions of the slider were a 217 mm diameter, 150 mm inner diameter, 65 mm axial thickness, and a wall thickness of 2 mm. The profile dimensions of the paddles were 65 mm × 50 mm × 2 mm.

**Figure 7:**
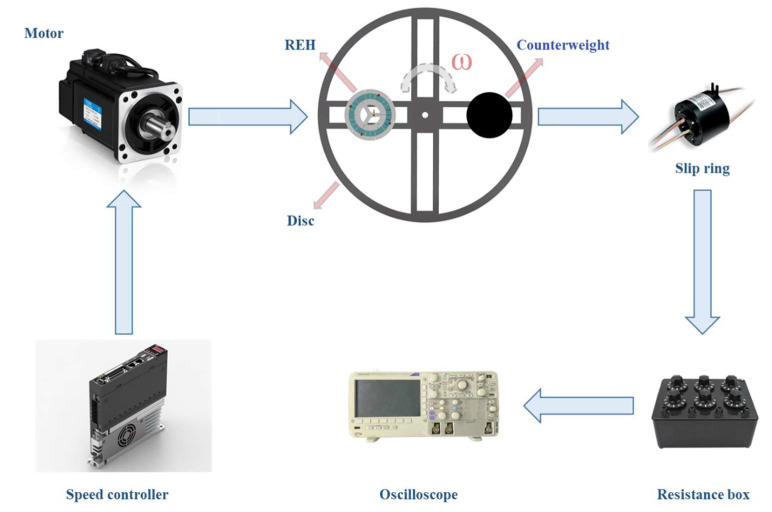
Experimental testing system for the REH.

To test the performance of the REH, a comprehensive testing system was established, as shown in Figure 7. The rotational excitation was provided by a servo motor, with the rotational speed controlled by a controller. The REH was installed on an acrylic disc with a diameter of 1200 mm, and the eccentricity distance of the REH was adjusted through an array of small holes on the disc. One REH and one counterweight were mounted on the left and right sides of the disc, respectively, and balanced using the lever principle to ensure the stable rotation of the entire system. The output (V_RMS_) of the piezoelectric beams inside the REH was measured and recorded by connecting wires through a slip ring to an oscilloscope (Keysight DSO-X 2024A, Keysight Technologies, Santa Rosa, CA, USA).

## 4. Results and Discussion

**Figure 8:**
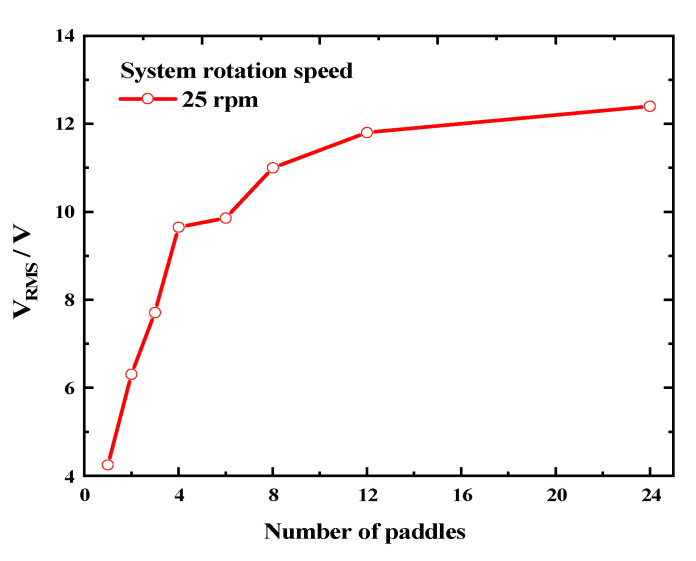
Variation in open-circuit voltage according to the number of paddles (system rotation speed *ω =* 25 rpm).

To investigate the feasibility of enhancing the output performance of a rotational energy harvester (REH) in ultra-low-frequency rotating environments by increasing the number of paddles, we tested the output characteristics of an REH with a piezoelectric beam under the experimental conditions of a system speed of 25 rpm and eccentricity distance of 160 mm. The V_RMS_ (root mean square of voltage) results are shown in Figure 8. It can be observed that as the number of paddles increased from 1 to 8, the effective output voltage of the REH rapidly rose from 4.25 V to 11 V. However, as the number of paddles further increased, the rising trend weakened. The open-circuit voltages obtained for 12 and 24 paddles were 11.8 V and 12.4 V, respectively. These represent only a 7.3% and 12.7% increment compared to REH-8, despite having 1.5 and 3 times more paddles, respectively. This phenomenon could be attributed to the decreasing time interval for the piezoelectric beam to be actuated as the number of paddles increased. This was manifested in the output waveform shown in Figure 3c, where with an increasing number of paddles, an overlap between adjacent output waveforms became apparent. This led to energy loss, thereby limiting the improvement in the REH’s output performance.

**Figure 9:**
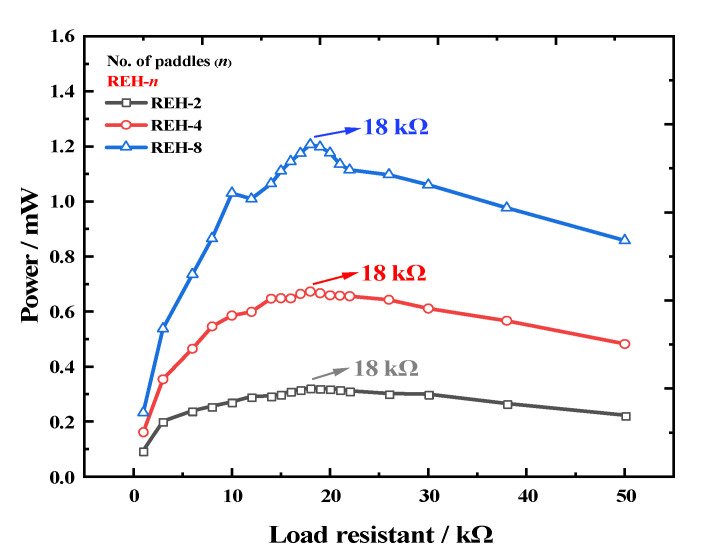
Output power of the REH as a function of load under different paddle numbers (system rotation speed *ω =* 25 rpm).

To further validate the relationship between the number of paddles and the output performance, we studied the variation in power with the number of paddles at the optimal load for the REH. First, under the experimental conditions of a system speed of 25 rpm and an eccentricity distance of 160 mm, we tested the optimal load characteristics of an REH with 2, 4, and 8 paddles. As shown in Figure 9, the optimal load (R) values for all three configurations were around 18 kΩ, indicating that the number of paddles did not alter the optimal load of the device.

**Figure 10:**
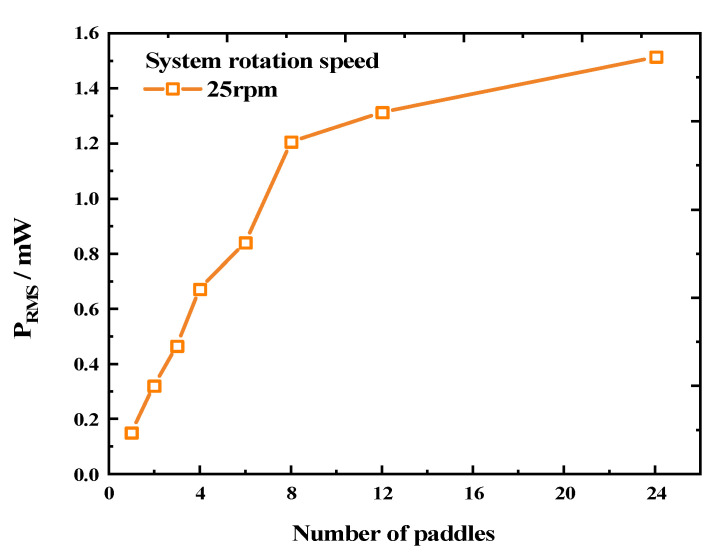
Variation in output power according to the number of paddles (system speed *ω =* 25 rpm).

Based on this, we proceeded to test the output power of the REH with different numbers of paddles at the optimal load. Figure 10 illustrates that as the number of paddles increased from 1 to 8, the P_RMS_ (root mean square of power) of the REH at the optimal load rose rapidly from 0.153 mW to 1.206 mW, reaching 7.9 times the initial output value. However, as the number of paddles further increased, the rising trend weakened. The output powers obtained for 12 and 24 paddles were 1.313 mW and 1.514 mW, respectively, representing only an 8.9% and 25.5% increment compared to REH-8. This changing trend was similar to the voltage variation trend shown in Figure 8, and the underlying reason was the same, thus avoiding repetition.

From the aforementioned experimental results, it is evident that the number of paddles had a significant impact on the output performance of the REH in the range of 1 to 8. To further validate this output characteristic, we investigated the effects of parameters such as rotational speed and eccentricity distance on the REH output performance while varying the number of paddles.

**Figure 11:**
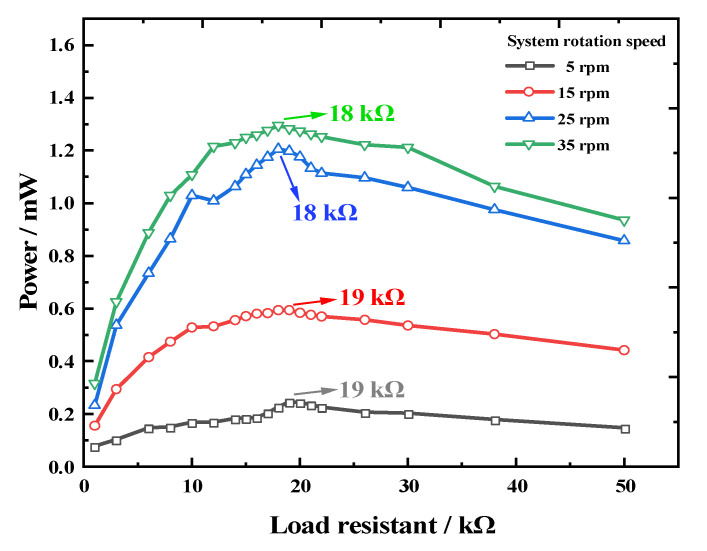
Output power of the REH as a function of load under different rotational speeds (number of paddles *n* = 8).

To explore the influence of the rotational speed on the REH output performance, we initially studied the optimal load characteristics of the REH at different speeds. Taking REH-8 as the test specimen, we performed measurements of the optimal load under four different speeds (5 rpm, 15 rpm, 25 rpm, and 35 rpm) when the eccentricity distance was set at 160 mm. The results shown in Figure 11 indicate that the optimal load values for the device remained around 19 kΩ for 5 rpm and 15 rpm conditions, while for 25 rpm and 35 rpm conditions, the optimal load values were around 18 kΩ. Thus, at lower rotational speeds, the REH’s optimal load remained between 18 kΩ and 19 kΩ.

**Figure 12:**
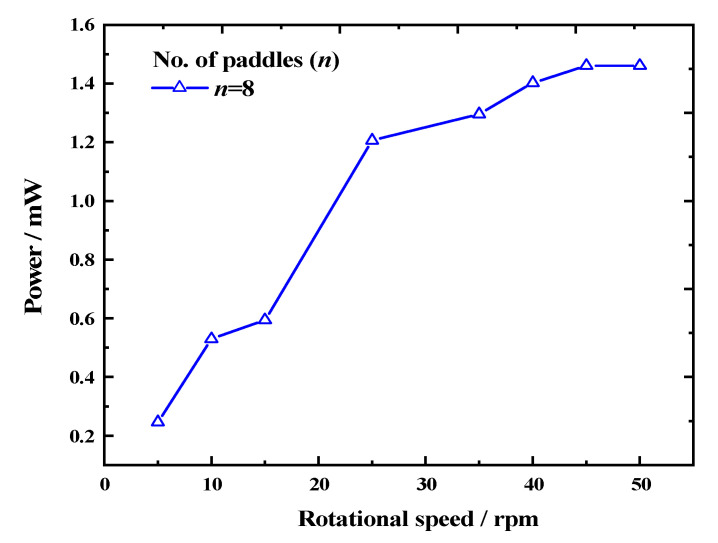
Variation in output power according to rotational speed (number of paddles *n* = 8).

Based on this, we conducted tests on the optimal output of the device at different speeds. Figure 12 demonstrates that as the speed increased, the REH’s output power at the optimal load continued to rise. As the speed increased from 5 rpm to 40 rpm, the REH’s output power increased from 0.246 mW to 1.402 mW, representing a 5.7-fold increment. However, as the speed further increased, the upward trend in output power weakened. At a speed of 50 rpm, the output power of the device was 1.461 mW, which was only a 4.2% increase compared to the output at 40 rpm. The possible cause for this trend could have been that as the system rotation speed increased, the frequency at which the piezoelectric beams were struck also increased, leading to mutual interference between adjacent strikes. This ultimately resulted in a decrease in the rising trend of the device’s output power, as demonstrated in the waveform shown in Figure 3c.

**Figure 13:**
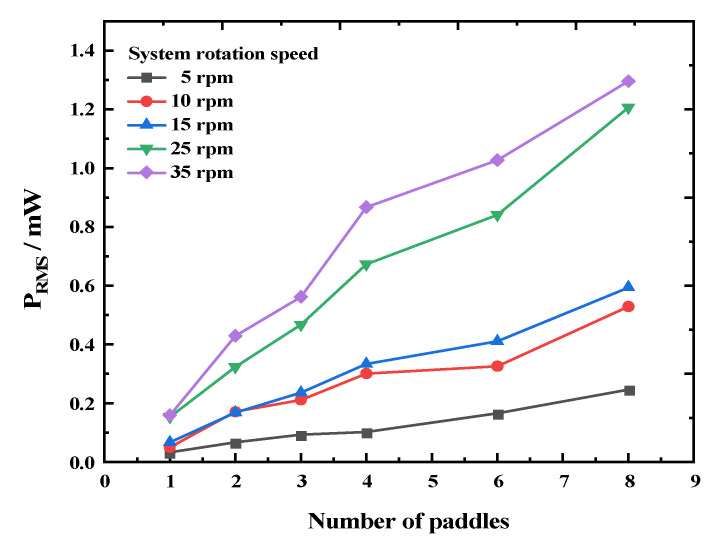
Output power of the REH as a function of the number of paddles under different rotational speeds.

**Figure 14:**
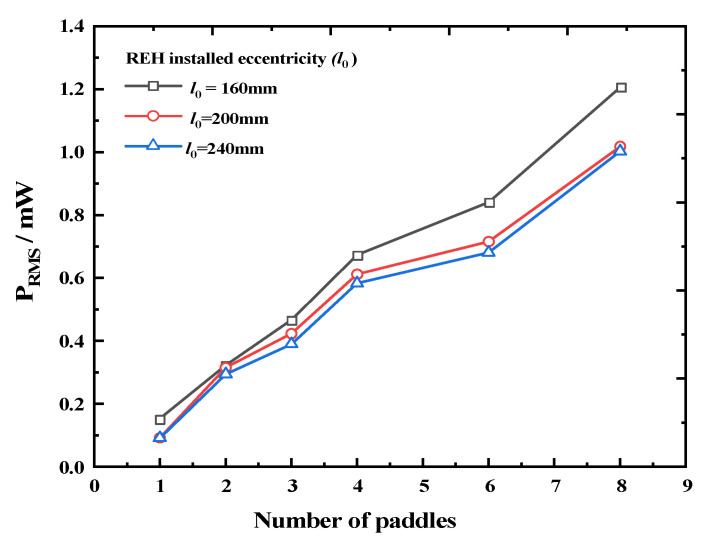
Output power of the REH as a function of the number of paddles under different eccentricities (system rotation speed *ω* = 25 rpm).

Additionally, we investigated the relationship between the output performance of the REH and the number of paddles under different rotation speeds and eccentricity conditions, and the experimental results are shown in Figure 13 and Figure 14. As shown in Figure 13, at different rotation speeds, the output power of the REH under the optimal load increased with an increasing number of paddles. Among them, REH-8 achieved a maximum power output of 1.296 mW at a speed of 35 rpm. Similarly, as shown in Figure 14, under different eccentricity conditions, the device’s output power also increased with an increasing number of paddles. Therefore, within a certain range of values, variations in rotation speed and eccentricity did not significantly affect the improvement in output performance due to the number of paddles.

**Figure 15:**
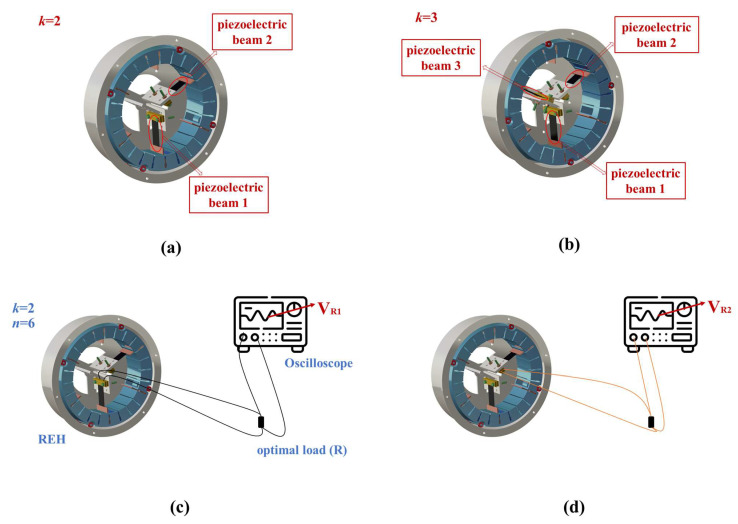
Multi-piezoelectric-beam REH model and output testing method: (**a**) the REH with an internal piezoelectric beam count of *k* = 2; (**b**) the REH with an internal piezoelectric beam count of *k* = 3; (**c**) the measurement of V_R_ for piezoelectric beam 1; (**d**) the measurement of V_R_ for piezoelectric beam 2.

**Figure 16:**
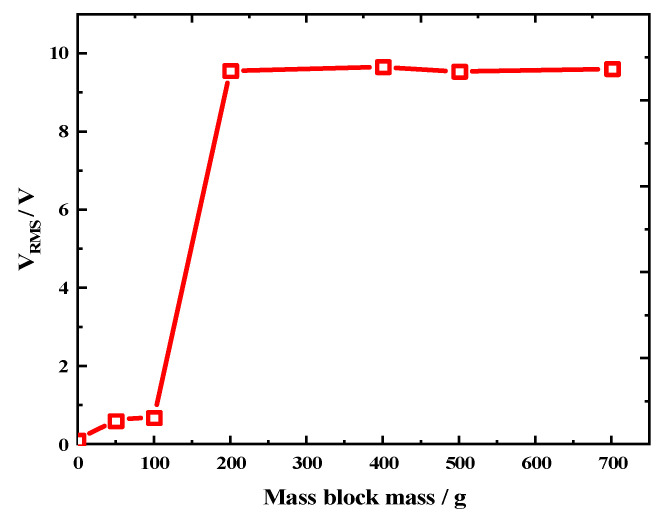
Variation in open-circuit voltage according to the mass block mass (system rotation speed *ω* = 30 rpm, number of paddles *n* = 8, number of piezoelectric beams *k* = 1).

Hence, we concluded that increasing the number of paddles was a feasible approach to enhancing the output performance of the REH. Furthermore, to further improve the output performance, we employed an array structure to increase the number of piezoelectric beams inside the REH. Figure 15a,b present schematic diagrams of the REH structure with *n* = 2 and *n* = 3, respectively. Simultaneously, to obtain the P_RMS_ of a multi-piezoelectric-beam REH, we sequentially measured the output voltage of each individual piezoelectric beam and calculated the output power (as shown in Figure 15c,d); then, we summed the power of each piezoelectric beam to obtain the total output power of the REH ($P_{RMS}=\sum_{x=1}^{k}\frac{V_{Rx}}{Rx}$, V_R_ represents the effective voltage across the optimal load (R)). However, frequent collisions could potentially result in a loss of kinetic energy for the slider, which might have undermined the effectiveness of individual impacts and subsequently impacted the output performance of the REH. As the kinetic energy of the slider was directly proportional to its mass when the velocity was constant, we investigated the impact of the mass block mass (*G*) on the output performance of the REH (system rotation speed *ω* = 30 rpm, number of paddles *n* = 8, number of piezoelectric beams *k* = 1), as depicted in Figure 16. When *G* ≤ 100 g, the paddles within the REH were unable to drive the piezoelectric beams into self-excited oscillations during its operational process. As a result, the slider and the shell remained relatively stationary during system rotation, leading to minimal external output from the REH. At *G* = 200 g, the paddles were able to induce self-excited oscillations in the piezoelectric beams, generating external output. However, as *G* was further increased (>200 g), the REH’s output did not exhibit a growth trend (V_RMS_ = (9.6 ± 0.5) V). This indicates that once the mass block mass was sufficient to initiate self-excited oscillations in the piezoelectric beams, the impact of *G* on the REH’s output became relatively minor.

**Figure 17:**
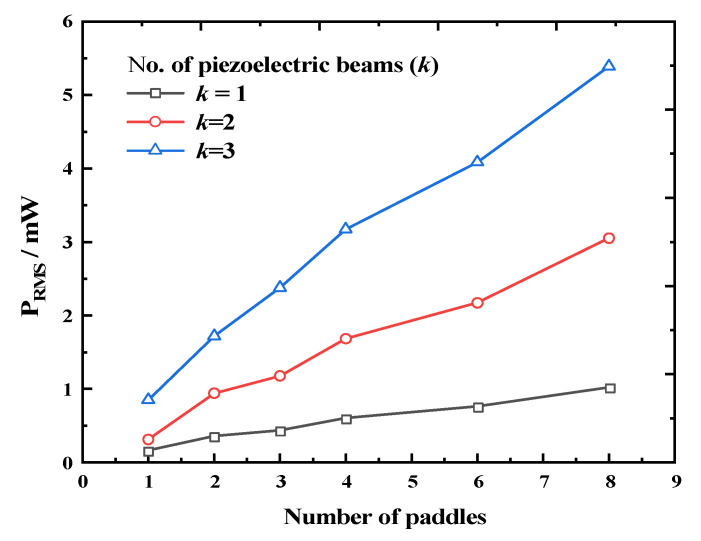
Output power of the REH as a function of the number of paddles under different numbers of piezoelectric beams (system rotation speed *ω* = 25 rpm).

Hence, we investigated the output characteristics of the REH with different numbers of piezoelectric beams when the mass of the quality block was sufficiently large (*G* = 500 g), as shown in Figure 17. For *k* = 1, the output power of the REH increased with an increasing number of paddles, reaching a maximum power output of 0.898 mW for REH-8. For *k* = 2, the REH achieved an output power of 3.052 mW under the same external conditions. With *k* = 3, the REH achieved an output power of 5.392 mW, with the highest output of an individual beam reaching 2.268 mW. This was achieved by adjusting the relative distance between the top of the piezoelectric beam and the top of the paddle, thereby increasing the contact area during the collision and enhancing the effectiveness of the impact between the paddle and the piezoelectric beam. Thus, it could be concluded that the proposed method of incorporating an array of piezoelectric beams into the REH was feasible for enhancing its output performance.

To evaluate the performance superiority of the designed REH, we compared it with existing energy harvesters, as shown in Table 3. The proposed REH achieved a maximum output power of 5.392 mW, demonstrating a significant advantage in output performance compared to other energy harvesters. Additionally, the maximum power density of the proposed REH reached 4.02 μW/(cm^3^ Hz), indicating substantial improvement in output performance compared to the device in our previous work [41], which had a power density of 0.23 μW/(cm^3^ Hz).

## 5. Conclusions

In this paper, a novel ultra-low-frequency rotational energy harvester based on a double-frequency up-conversion mechanism was proposed, enabling the efficient harvesting of rotational energy in ultra-low-frequency rotational environments (<1 Hz) and demonstrating satisfactory output performance. The proposed rotational energy harvester (REH) consisted of a cylindrical shell with an array of piezoelectric beams and a slider with multiple paddles. During operation, the relative motion between the slider and the shell caused the paddles to periodically strike the piezoelectric beams, inducing self-excited oscillation in the beams (the first frequency up-conversion) and converting mechanical energy into electrical energy through the piezoelectric effect. Additionally, by increasing the number of paddles on the slider and the piezoelectric beams inside the REH, the frequency at which the beams were struck during one rotation cycle could be further increased (the second frequency up-conversion), enhancing the output performance of the REH. Experimental investigations were conducted to study the relationship between the output performance of the proposed REH and parameters such as the number of paddles and the system rotation speed. The data showed that under low-frequency rotation and low-eccentricity conditions, the output performance of the proposed REH could be improved by increasing the number of paddles and the system rotation speed. Furthermore, to further enhance the output performance of the REH, an array of piezoelectric beams was introduced inside the shell, and the output performance of the REH with different numbers of piezoelectric beams was tested. The experimental results revealed that the maximum output power of an individual piezoelectric beam reached 2.268 mW, while the REH with three piezoelectric beams achieved an output power of 5.392 mW, with a power density of 4.02 μW/(cm^3^ Hz) under a rotational excitation of 0.42 Hz, exhibiting better output performance compared to similar REHs.

It is worth noting that the main purpose of this study was to demonstrate the advantages of the proposed REH structural design based on a double-frequency up-conversion mechanism in terms of performance enhancement. However, in order to further enhance and evaluate the output performance of the proposed REH, the optimization of the REH design parameters (such as the number of piezoelectric beams, the number of paddles, the eccentricity, and the slider mass) should also be investigated. Furthermore, practical application-related performance testing and the design of energy management circuits must be considered. These issues will be addressed in our future research.

## Figures and Tables

**Table 1:** Relevant parameters in Figure 4.

Symbols	Meaning
*ω*	System angular velocity
*t*	System motion time
*l*_0_	Installation eccentricity of the REH
*l*	Eccentricity of the slider’s centroid
*a*	Acceleration of the slider relative to the shell
*g*	Gravitational acceleration
*θ*	Angular displacement of the centroid relative to the center of the shell
*θ*_1_	Angular displacement of the centroid relative to the center of rotation
*θ*_2_	*θ* − *θ*_1_
*F_N_*	Support force from shell
*F_c_*	Centrifugal force of slider
*m*	Mass of slider
*R*	Radius of the cylindrical shell
*r*	Distance from the centroid of the slider to the bottom of the shell
*F_k_*	Coriolis force of slider
*F_f_*	Frictional force

**Table 2:** Parameters of the beam structure and piezoelectric elements (PZT 5J S230-J1FR-1808XB, Made Technology Co., Woburn, MA, USA).

Parameter	Value
Length of beam	55.3 mm
Width of beam	23.3 mm
Thickness of beam	0.46 mm
Length of PZT 5J	46 mm
Width of PZT 5J	20.8 mm
Thickness of PZT 5J	0.15 mm
Resonant frequency	130 Hz
Spring constant	0.25 N/mm

**Table 3:** A comparison of the proposed REH and typical REHs at a low rotational speed.

Reference	Number of Piezoelectric Beams	Frequency (Hz)	Power (μW)	Volume ^a^ (cm^3^)	Power Density (μW/(cm^3^ Hz)
[29]	1	3	2342	990	0.79
[39]	2	0.79	106	130.8	0.81
[43]	12	3.3	613	45.4	2.28
[41]	1	1.25	141	456.2	0.23
This work	1	0.42	2268	3185.1	1.69
This work	3	0.42	5392	3185.1	4.02

^a^ Space demanded by the energy harvester during motion.

## Data Availability

Not applicable.
